# A New Membrane Lipid Raft Gene SpFLT-1 Facilitating the Endocytosis of *Vibrio alginolyticus* in the Crab *Scylla paramamosain*

**DOI:** 10.1371/journal.pone.0133443

**Published:** 2015-07-17

**Authors:** Fangyi Chen, Jun Bo, Xiaowan Ma, Lixia Dong, Zhongguo Shan, Qian Cui, Huiyun Chen, Kejian Wang

**Affiliations:** 1 State Key Laboratory of Marine Environmental Science, College of Ocean & Earth Science, Xiamen University, Xiamen, Fujian, P. R. China; 2 Fujian Collaborative Innovation Center for Exploitation and Utilization of Marine Biological Resources, Xiamen University, Xiamen, Fujian, P. R. China; 3 Fujian Engineering Laboratory of Marine Bioproducts and Technology, Xiamen University, Xiamen, Fujian, P. R. China; Boston Children's Hospital Harvard medical School Pediatrics, UNITED STATES

## Abstract

Pathogens can enter their host cells by way of endocytosis in which the membrane lipid raft gene flotillins are probably involved in the invasion process and this is an important way to cause infection. In this study, a new gene SpFLT-1 was identified in *Scylla paramamosain*, which shared high identity with the flotillin-1 of other species. The SpFLT-1 gene was widely distributed in tissues and showed the highest level of mRNA transcripts in the hemocytes. This gene might be a maternal gene based on the evident results that it was highly expressed in maternal ovaries and in the early developmental stages of the zygote and early embryo stage whereas it gradually decreased in zoea 1. SpFLT-1 positively responded to the challenge of *Vibrio alginolyticus* with a significantly increased level of mRNA expression in the hemocytes and gills at 3 hours post infection (hpi). The SpFLT-1 protein was detected densely in the same fraction layer where the *Vibrio* protein was most present in the hemocytes and gills at 3 hpi. Furthermore, it was found that the expression of SpFLT-1 decreased to the base level following disappearance of the *Vibrio* protein at 6 hpi in the gills. Silencing SpFLT-1 inhibited the endocytosis rate of *V*. *alginolyticus* but overexpression of the gene could facilitate bacterial entry into the epithelioma papulosum cyprinid cells. Our study indicated that SpFLT-1 may act as a key protein involved in the process of bacterial infection and this sheds light on clarifying the pathogenesis of pathogens infecting *S*. *paramamosain*.

## Introduction

The mud crab, *Scylla paramamosain*, is one of the most important marine breeding crabs in China, with vital nutritional and economic value. The animals are usually raised in ponds at high densities, which has led to disease epidemics in recent years. Bacteria, especially the Gram-negative bacteria (e.g. *Vibrio*), are one of the leading pathogens infecting these crabs. However, the pathogenesis is poorly understood and as yet there are no effective immune control measures. Therefore, clarifying the pathogenic invasion pathways of the pathogens into host cells will give us a direction in future as to how to control the disease outbreak in crab aquaculture.

Invertebrate animals lack an adaptive immune system and they have to rely on their innate immune system in which immune-related components may play a key role [1]. As with other invertebrates, a number of innate immune-related genes have been identified so far from *S*. *paramamosain*, including antimicrobial peptides [2–4] and pathogen recognition protein [5, 6], which are found to have potentially critical roles in defense against pathogens. To evaluate more components, a subtractive suppression hybridization (SSH) cDNA library was constructed and many interesting genes or proteins potentially involved in the innate immune defense of the crab were screened from the hemocytes of the crab *S*. *paramamosain* challenged with lipopolysaccharide (LPS) [7]. Among them, a new membrane lipid raft gene flotillin-1 was identified which was possibly involved in the pathogenesis of invading pathogens, and thus worth further study.

Flotillins, also named as reggies, are considered as scaffold proteins in lipid rafts and marker proteins of lipid microdomains. The flotillin protein family consists of two highly conserved molecules, flotillin-1 and -2. Beyond their functions in signaling [8, 9], endocytosis [10], interactions with the cytoskeleton [11], and cell proliferation [12], recent studies have shown that flotillins may be involved in the process of pathogenic infection in mammalian cells. Flotillins are crucial for intracellular trafficking of cholera, Shiga and ricin toxin and would affect their toxicity [13, 14]. The flotillin family is also associated with several human diseases [15–18]. Flotillin-1 was found to contribute to the intracellular growth of *Chlamydia pneumoniae* [19]. In addition, studies reveal that flotillin-1 is enriched in the mature phagosome and plays a specific function in the process of phagolysosome formation, which may prevent the growth of intracellular pathogens [20]. In our study, the flotillin-1 which is screened in *S*. *paramamosain* was named SpFLT-1. It is the first time that this gene has been reported in crustaceans. Because of its up-regulation in LPS-challenged crabs, SpFLT-1 was predicted to be involved in the immune defense process of *S*. *paramamosain*. However, until now, the information on SpFLT-1 is not well known and it is still not clear whether any interaction is exhibited between SpFLT-1 and bacteria in crabs as it is in human beings, and whether it could assist bacterial invasion into the host cells or effectively inhibit the toxicity of intracellular bacterial toxins.

Following on from previous work, a full-length cDNA of SpFLT-1 gene was cloned and its genomic DNA organization was characterized. The tissue distribution and mRNA expression of this gene in embryonic development stages were also investigated in *S*. *paramamosain*. To reveal whether SpFLT-1 gene responded to *Vibrio* infection, the expression profile and potential interaction between SpFLT-1 and *Vibrio* protein in the hemocytes and gills after *Vibrio alginolyticus* challenge were determined using relative quantitative real-time PCR (qPCR) and immune-blotting. In addition, the endocytosis of *V*. *alginolyticus* was further studied using the SpFLT-1 RNA interference (RNAi) assay and overexpression detected using confocal microscopy and the iCys quantitative image cytometer.

## Results

### Isolation and sequence analysis of SpFLT-1

**Fig 1:**
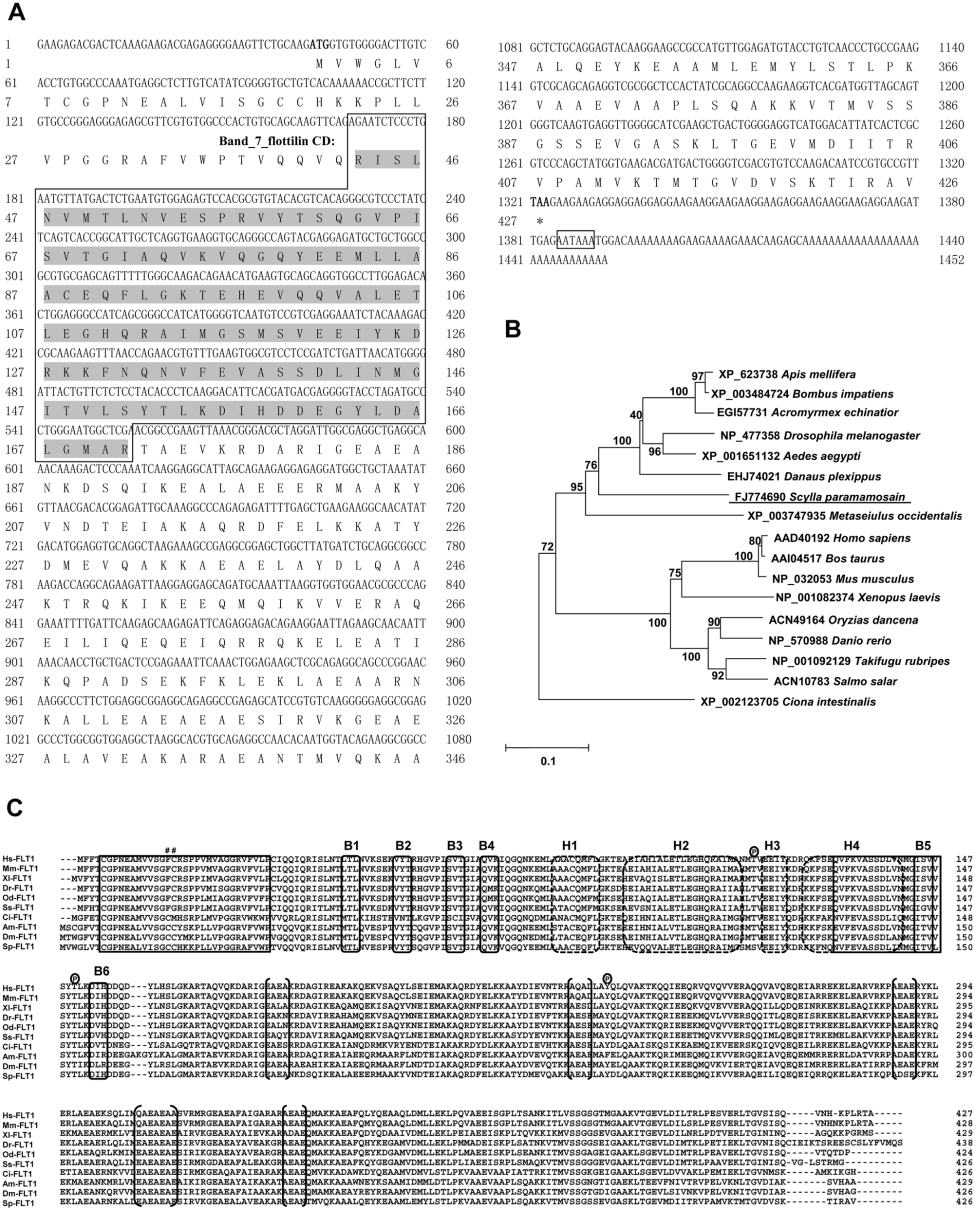
(A) Complementary DNA and predicted amino acid sequences of SpFLT-1 from *S*. *paramamosain*. The putative conserved domain of the flotillin/SPFH family is framed gray highlighted. The polyadenylation signal is boxed and the stop codon is indicated by “*”. (B) Phylogenetic analysis of deduced amino acid sequences from SpFLT-1 and the flotillin-1 of other species. SpFLT-1 is underlined. Numbers next to the branches indicate bootstrap value of each internal branch in the phylogenetic tree nodes from 1000 replicates. (C) Multiple alignment of amino acid sequences among SpFLT-1 and the flotillin-1 of other species. The pond sign and enclosed Pabove the alignment indicate the putative palmitoylation and phosphorylation sites. Secondary-structure predictions: six β strand (B1 to B6) and five α helices (H1 to H5) are accordingly shown in the solid and dashed line rounded rectangle. Both conserved N-terminal hydrophobic stretches are in framed boxes. EA-rich coiled-coil regions are designated in parentheses. Amino acid residues are numbered to the right of each sequence. Flotillin-1 amino acid sequences were obtained from GenBank as follows, Hs-FLT1: *H*. *sapiens* (human) (AC AAD40192); Mm-FLT1: *M*. *musculus* (house mouse) (AC NP_032053); Xl-FLT1: *X*. *laevis* (African clawed frog) (AC NP_001082374); Dr-FLT1: *D*. *rerio* (zebrafish) (AC NP_570988); Od-FLT1: *O*. *dancena* (brackish medaka) (AC ACN49164); Ss-FLT1: *S*. *salar* (Atlantic salmon) (AC ACN10783); Ci-FLT1: *C*. *intestinalis* (vase tunicate) (AC XP_002123705); Am-FLT1: *A*. *mellifera* (honey bee) (AC XP_623738); Dm-FLT1: *D*. *melanogaster* (fruit fly) (AC NP_477358); Sp-FLT1: *S*. *paramamosain* (AC FJ774690).

A SpFLT-1 cDNA of 1452 nucleotides (GenBank accession number (AC) **FJ774690**), with an open reading frame (ORF) of 1278 bp was obtained using rapid amplification of cDNA ends (RACE) PCR (Fig 1A). It encodes a polypeptide of 426 amino acid residues with an estimated molecular mass of 47 kDa and an isoelectric point of 5.3.

Phylogenetic relationship analysis indicated that the selected flotillin-1 sequences were divided into two major groups. SpFLT-1 clustered into invertebrate clades and was closely positioned to *Aedes aegypti* and *Drosophila melanogaster*. In the vertebrate cluster, there were various mammalians and fishes as shown in Fig 1B.

Multiple sequence alignment of the deduced amino acid sequences of SpFLT-1 revealed that it was highly evolutionarily conservative from mammals to invertebrates. In the currently known flotillin-1, SpFLT-1 has the highest identity to yellow fever mosquito (*A*. *aegypti*), fruit fly (*D*. *melanogaster*), and honey bee (*Apis mellifera*) with identity to 74 or 75%. It has 63% sequence identity with the zebrafish (*Danio rerio*). In comparison with mammalians, such as the house mouse (*Mus musculus*) and humans (*Homo sapiens*), they share 62% amino acid sequence identity (Fig 1C and S2 Table).

Homologous sequence alignment demonstrated that all flotillin-1 possessed the putative palmitoylation and phosphorylation sites. The secondary structure predictions showed that there were six β strands and five α helices. Two conserved N-terminal hydrophobic stretches were observed and the EA-rich coiled-coil regions existed in the C-terminal of flotillin-1 (Fig 1C).

**Fig 2:**
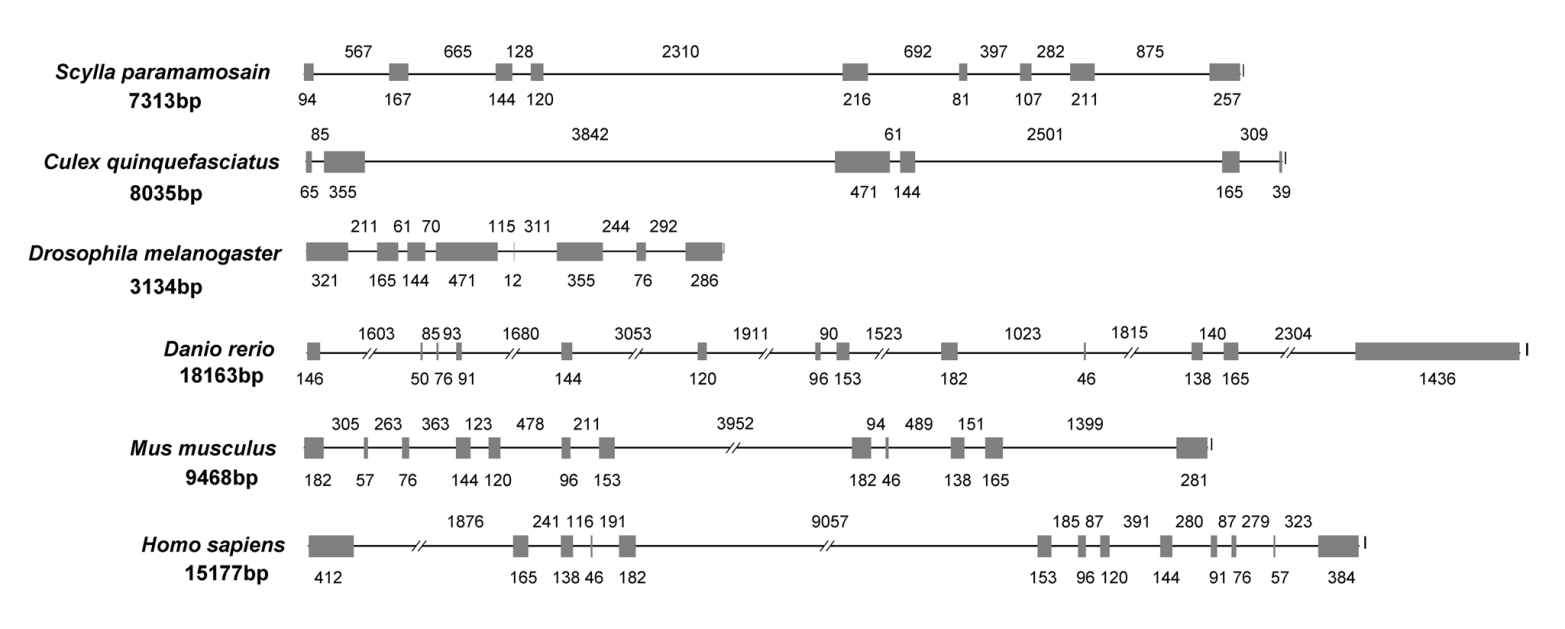
Genomic organization of flotillin-1. Comparison of flotillin-1 gene structure among *S*. *paramamosain* (AC **JX228176**), *C*. *quinquefasciatus* (southern house mosquito) (AC **NW_001886902**), *D*. *melanogaster* (fruit fly) (AC **NT_033778**), *D*. *rerio* (zebrafish) (AC **NC_007130**), *M*. *musculus* (house mouse) (AC **NC_000083**) and *H*. *sapiens* (human) (AC **NC_000006**). Exons are indicated by boxes and introns by lines. The numbers below the boxes and above the lines indicate length in base pairs.

The full-length of the SpFLT-1 genomic DNA consisted of 7313 bp (AC **JX228176**) and contained nine exons and eight introns. All introns were located within the ORF and the exon—intron junctions followed the consensus rule of the splice acceptor—AG/GT—splice donor for splicing. The precise lengths of the exons and introns of SpFLT-1 were compared with those of vertebrates and invertebrates (Fig 2). The genomic organization of SpFLT-1 was different from that of humans (AC **NC_000006**), the house mouse (AC **NC_000083**), zebrafish (AC **NC_007130**), fruit fly (AC **NT_033778**) and southern house mosquito (AC **NW_001886902**). The exons and introns of flotillin-1 in invertebrates (about 6–9 exons) were less than those in vertebrates (about 13 exons) and the length of the genomic DNA was the same.

### The expression pattern of SpFLT-1 in different tissues and embryonic development stages

**Fig 3:**
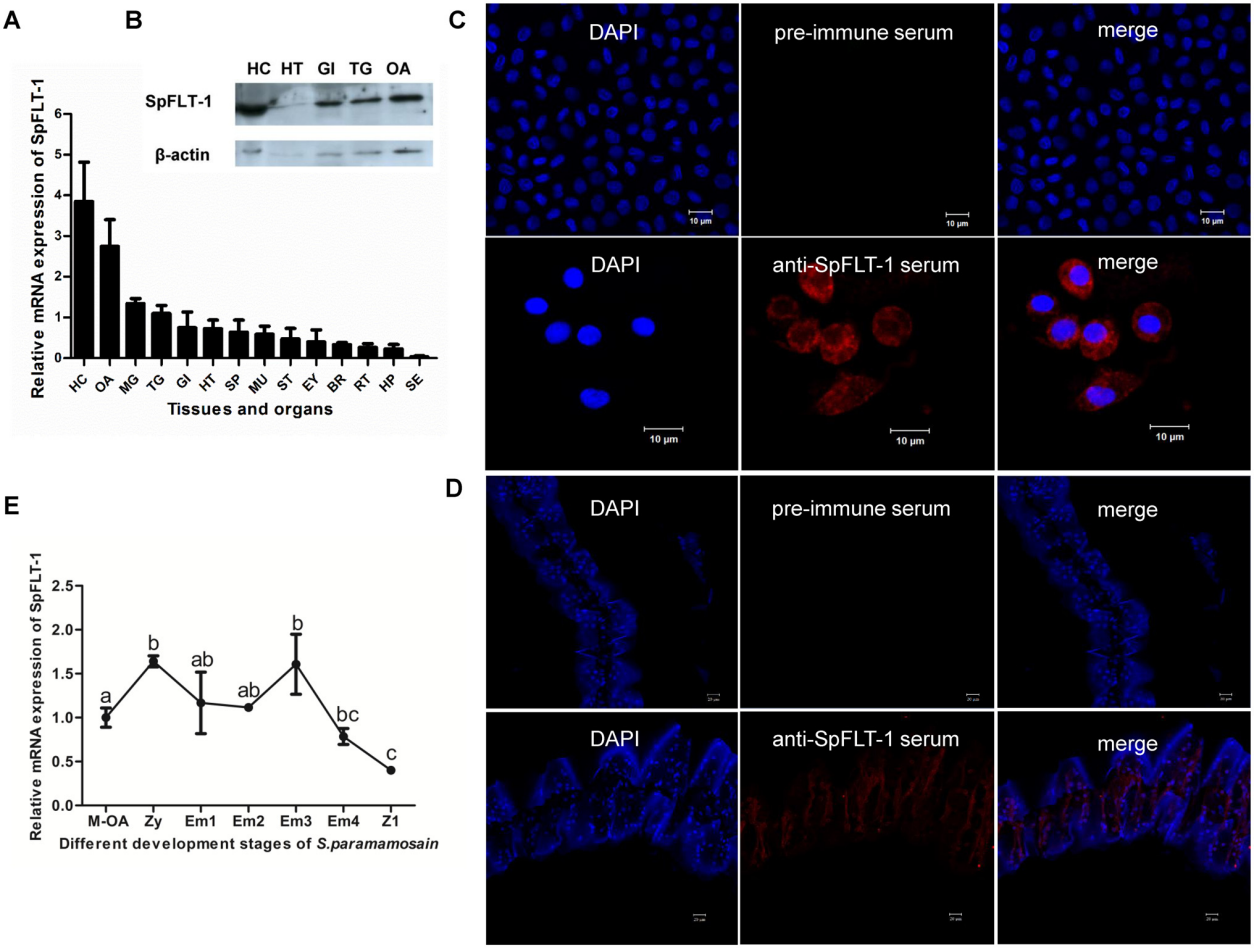
Expression of SpFLT-1 in different tissues and embryonic development stages from *S*. *paramamosain*. (A) Relative gene expression of SpFLT-1 in tissues. HC: hemocytes; OA: ovaries; MG: midgut gland; TG: thoracic ganglion mass; GI: gills; HT: heart; SP: spermathecae; MU: muscle; ST: stomach; EY: eyestalk; BR: brain; RT: reproductive tract; HP: hepatopancreas; SE: subcuticular epithelia. (B) Immune-blotting analysis of SpFLT-1 protein expression in selected tissues; (C & D) Immunofluorescence analysis of SpFLT-1 protein in HC and GI. SpFLT-1 protein was detected with an antibody specific for SpFLT-1 and anti-mouse Alexa Fuluor 647-conjugated secondary antibody and is shown in red. Pre-immune serum was used as a control. DNA in HC and GI were stained with DAPI and are shown in blue. Bar, HC: 10 μm, GI: 20 μm. (E) Relative gene expression of SpFLT-1 in maternal ovaries, zygote, early to late-staged embryos and the zoea 1 stage larva of *S*. *paramamosain*. M-OA: maternal ovaries; Zy: zygote; Em1, 2, 3, 4: embryo stages from early to late 1–4; Z1: zoea 1.

The qPCR results showed that SpFLT-1 gene had broad tissue distributions with the highest level of transcripts in the hemocytes, but moderate transcription in the ovaries, midgut gland, thoracic ganglion mass, gills and heart (Fig 3A). Prokaryotic expression and purification of SpFLT-1 recombinant protein and the specificity and high titer polyclonal antibodies were prepared as shown in S1 Fig. A similar expression pattern of SpFLT-1 protein was observed in the five tissues selected (Fig 3B). The subcellular localization of the SpFLT-1 in the hemocytes and gills was analyzed using confocal microscopy. The results showed that SpFLT-1 protein was distributed not only in the cell membrane but also probably in the cytoplasm of the hemocytes and it might be also expressed in gill epithelium cells (Fig 3C and 3D).

The transcripts of SpFLT-1 from maternal ovaries, zygote, early to late-staged embryos and zoea 1 stage larva of *S*. *paramamosain* were examined. SpFLT-1 showed high expression level in the zygote and early embryo stages similar in the maternal ovaries and gradually decreased to the lowest level in the zoea 1 stage (Fig 3E).

### The temporal expression pattern of SpFLT-1 post bacterial challenge

**Fig 4:**
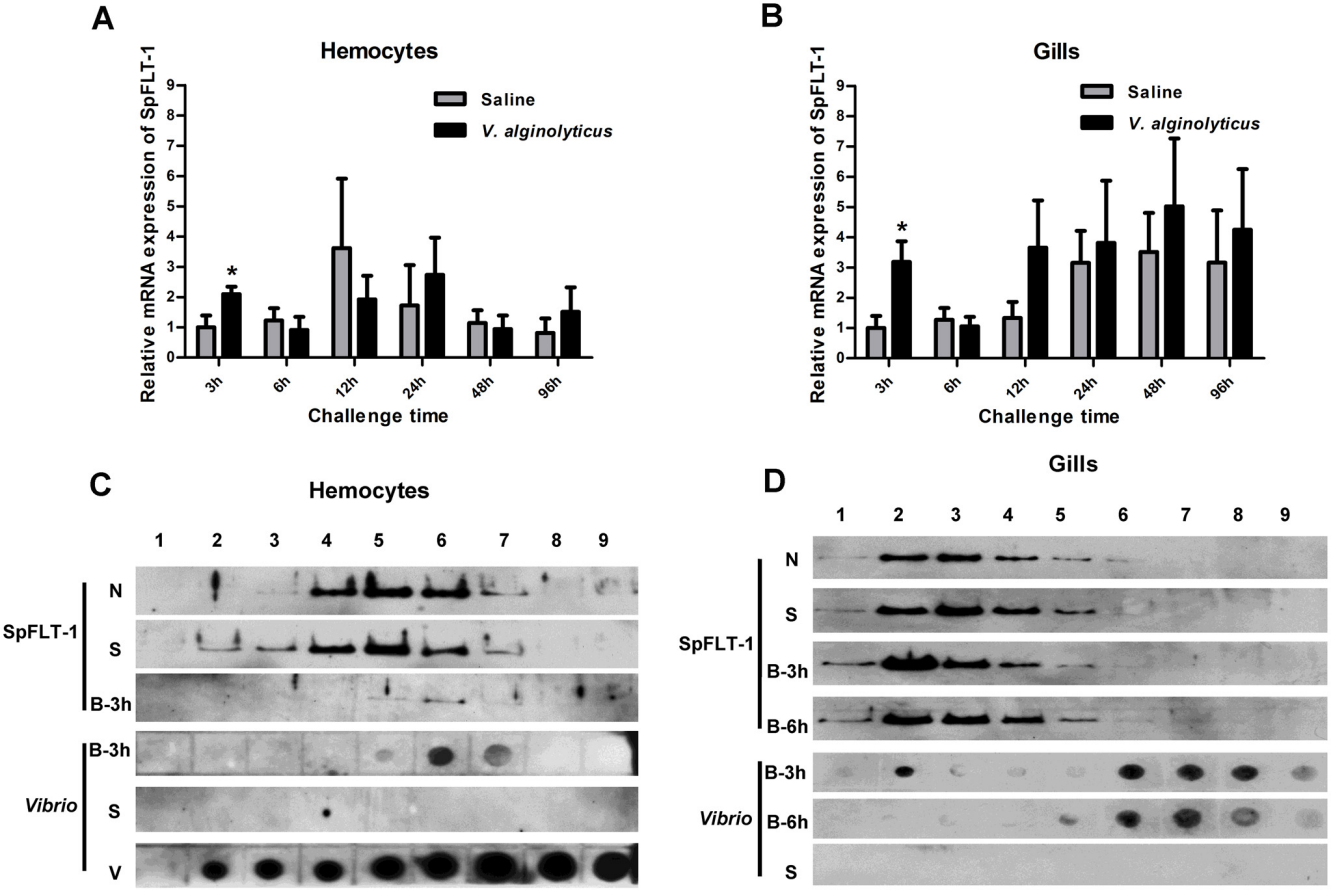
The expression profile of SpFLT-1 mRNA in hemocytes (Fig 4A) and gills (Fig 4B) post bacterial challenge using qPCR. The significant difference of SpFLT-1 transcripts between experimental group and control group is indicated with asterisks (*: *p* < 0.05). Bars indicate mean ±S.E. (n = 5). Immune-blotting analysis of expression of SpFLT-1 and *Vibrio* protein in hemocytes (Fig 4C) and gills (Fig 4D). N: normal group crabs; S: saline group crabs; B-3 h, B-6 h: crabs from 3 hpi or 6 hpi; V: *V*. *alginolyticus* alone. The figure is representative of results from three independent assays.

The SpFLT-1 gene was significantly up-regulated in hemocytes and gills at 3 h (2.1-fold and 3.2-fold, *p* < 0.05) post *V*. *alginolyticus* challenge (Fig 4A and 4B). We also tested whether the *Vibrio* protein was localized to the detergent-resistant membranes (DRMs) together with SpFLT-1. Our results showed that SpFLT-1 protein was localized in the same fraction layer (5, 6 and 7) as *Vibrio* protein in hemocytes at 3 hours post infection (hpi, Fig 4C). The total hemocyte counts of the crabs were dramatically decreased during the early stage after challenge with bacteria (data not shown), therefore the protein concentrations were relatively lower than in the control groups. In gills, the SpFLT-1 protein expression was higher in fraction 2 at 3 hpi which decreased at 6 hpi to the basal level, together with the *Vibrio* protein distributed in this same layer at 3 hpi, but disappeared at 6 hpi (Fig 4D).

### Silencing of SpFLT-1 inhibits the endocytosis rate of *V*. *alginolyticus*

**Fig 5:**
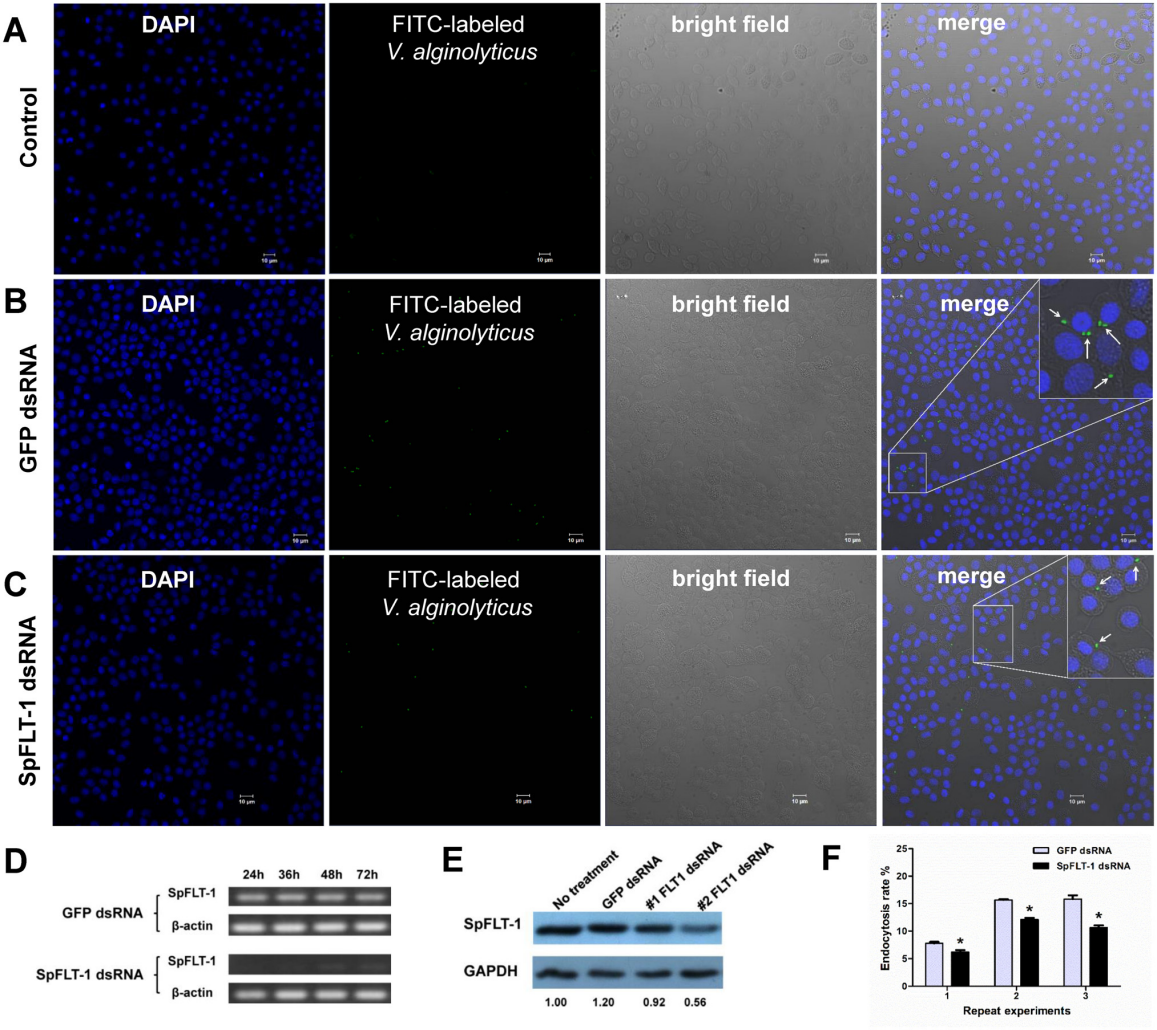
Silencing of SpFLT-1 inhibits the endocytosis rate of *V*. *alginolyticus* in the primary cultured hemocytes from *S*. *paramamosain*. (A) & (B) & (C) Representative images of endocytosis of *V*. *alginolyticus* in the primary cultured hemocytes treated by 4°C, GFP dsRNA or SpFLT-1 dsRNA using confocal microscopy. DNA in the hemocytes was stained with DAPI and is shown in blue. FITC-labeled *V*. *alginolyticus* is green. Bar: 10 μm. (D) & (E) SpFLT-1 expression was analyzed using semi-quantitative PCR (left) and immune-blotting (right) in the RNA interference assays. GAPDH was used as a loading control. (F) The endocytosis rates of the cells were determined using an iCys quantitative image cytometer. Data from three independent experiments were analyzed. Bars indicate mean ± S.E.

To evaluate the role of SpFLT-1 in the endocytosis of *V*. *alginolyticus*, double stranded RNA (dsRNA) was employed to target the SpFLT-1 gene in the primary cultured hemocytes of *S*. *paramamosain* and endocytosis assay was carried out by counting the bacteria ingested in the hemocytes. The efficiency of RNAi-mediated transcript depletion was determined using semi-quantitative RT-PCR. One of the representative results showed that the SpFLT-1 mRNA transcript was reduced to ~47% at 24 hpi and ~36% at 36 hpi (Fig 5D). Also, the SpFLT-1 protein expression was decreased to 0.56 in the alternative number 2 dsRNA group at 36 hpi which is the highest interference efficiency time point (Fig 5E).

The representative images of 4°C (control), GFP and SpFLT-1 dsRNA treated cells infected with FITC-conjugated *V*. *alginolyticus* are shown in Fig 5A, 5B and 5C. Millions of cells including the cells with ingested fluorescent bacteria were counted. The endocytosis rate was significantly decreased in the SpFLT-1 dsRNA group cells (Fig 5F).

### SpFLT-1 facilitates the endocytosis of *V*. *alginolyticus* in EPC cells

**Fig 6:**
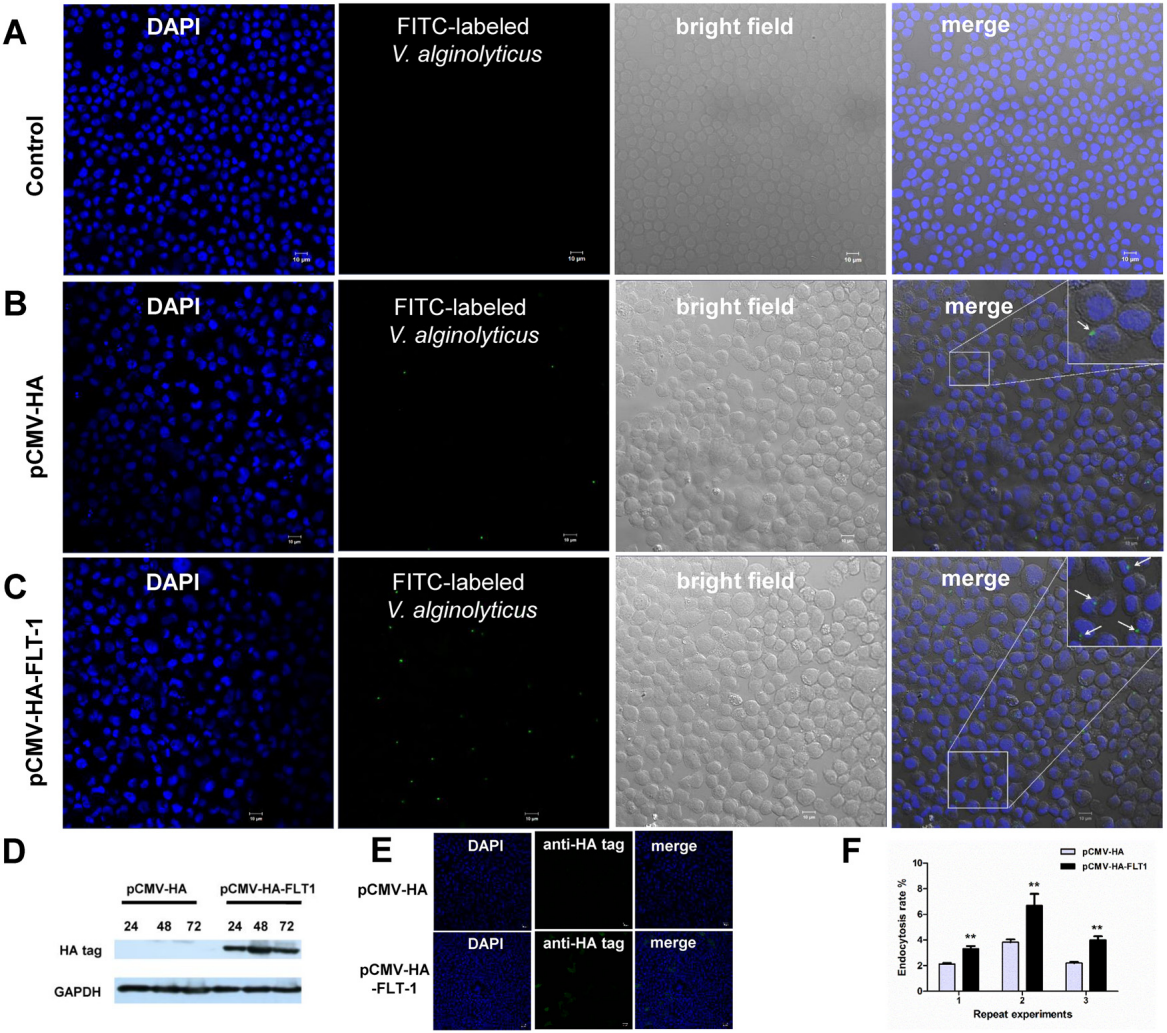
SpFLT-1 facilitates the endocytosis of *V*. *alginolyticus* in EPC cells. (A) & (B) & (C) Representative images of endocytosis of *V*. *alginolyticus* in 4°C treated EPC cells and cells transfected by pCMV-HA vector or pCMV-HA-FLT1 using confocal microscopy. DNA in the EPC cells was stained with DAPI and is shown in blue. FITC-labeled *V*. *alginolyticus* is green. Bar: 10 μm. (D) Immune-blotting analysis of expression of SpFLT-1 in EPC cells. GAPDH was used as a loading control. (E) Representative images of SpFLT-1 expression in pCMC-HA vector or pCMV-HA-FLT1 transfected cells using immunofifluorescence assay. SpFLT-1 protein detected with an antibody specific for HA-tag and anti-rabbit IgG FITC-conjugated secondary antibody is shown in green. DNA in the EPC cells was stained with DAPI and is shown in blue. Bar: 20 μm. (F) The endocytosis rates of the cells were determined using an iCys quantitative image cytometer. Data from three independent experiments were analyzed. Bars indicate mean ± S.E.

To further study the role of SpFLT-1 in the endocytosis of *V*. *alginolyticus*, pCMV-HA-FLT1 overexpression of the crab SpFLT-1 was generated. The immune-blotting results showed that pCMV-HA-FLT1 was successfully expressed in epithelioma papulosum cyprinid (EPC) cells from 24 h to 72 h post transfection with the highest expression at 48 hpi (Fig 6D). The immunofluorescence staining results also revealed that pCMV-HA-FLT1 was distributed in the EPC cells (Fig 6E). Overexpression of SpFLT-1 in the EPC cells could significantly increase the endocytosis rate of *V*. *alginolyticus* (Fig 6F).

## Discussion

Flotillin-1 has been implicated to be involved in a variety of different cellular processes, including endocytosis, signal transduction, regulation of cortical cytoskeleton, nerve cell regeneration and cell proliferation; and may play a role during pathogenic infection. To date, the molecular mechanisms of bacterial infection via endocytosis through the plasma membrane into host cells is presented in many publications [21–25], but fewer studies related to the endocytosis mechanism have been reported in marine invertebrates including crabs. In the present study, the finding of a new membrane lipid raft gene SpFLT-1 attracted us to questioning whether a similar endocytosis mechanism was exhibited in crabs to that confirmed in mammals.

The crab SpFLT-1 shows high evolutionary conservation which makes its protein structural features similar to the flotillin-1 of other species. It does not belong to the secreted protein and has no trans-membrane domain, but it could be inserted inside the cell membrane through its acylation sites with a predicted hydrophobic region at the N-terminal of the SPFH domain [26]. Therefore, the deduced SpFLT-1 palmitoylation sites in cysteine 8, 19 and 20 and two hydrophobic extensions may be required for subcellular localization of the SpFLT-1 (Fig 1C). In the C-terminal of SpFLT-1, there is a unique flotillin domain in which we could find several EA repeats (Fig 1C). These repeats can form a coiled-coil structure which may mediate the formation of stable homologous and heterologous polymers [27]. Coassembly of flotillins can induce the formation of membrane microdomains, membrane curvature, and vesicle budding [28, 29]. Previous studies show that phosphorylation of flotillins is regulated by the Fyn and Src kinase family mainly through their tyrosine residues, which affect the distribution and endocytosis of flotillin microdomains [30, 31]. The predicted phosphorylation sites of the SpFLT-1 protein were serine 119, threonine 153 and tyrosine 241 and they are critical for the functioning of SpFLT-1. In view of its highly conserved evolution, it could be speculated that the structure of the SpFLT-1 protein would provide a valuable reference for further uncovering the function of the *Scylla* flotillins protein.

As reported, flotillin-1 is widely expressed in mammalian tissues, being especially enriched in the nervous systems, adipose tissue, muscle, and erythrocytes [32–34]. The expression level of flotillin-1 is relatively high in blood cells, dendritic cells and dorsal root ganglion cells in humans and mice [35]. In our study, the SpFLT-1 gene was also found extensively distributed in various tissues and this indicated that it may play an important role in normal physiological processes in *S*. *paramamosain*. The highest expression of SpFLT-1 in the hemocytes and moderate expression in the gills of crabs suggested that it may be directly involved in immune defense and its function possibly associated with the bacterial infection process.

Previous studies confirm that flotillins are mainly localized in the plasma membrane of many cell types, such as nerve cells and lymphocytes [36]. In addition to the distribution in the plasma membrane, flotillins are found in a variety of intracellular vesicles. Studies report that, in some cell types, flotillins, especially flotillin-1, are also localized in endosomes [37], lysosomes [38], phago-lysosomes [20], multi-vesicular bodies [39] and exosomes [40]. Flotillins are also found existing at the Golgi apparatus [41] and after binding to phosphatase PTOV-1, flotillin-1 can be located in the nucleus [42]. Similarly, they are distributed in lipid-enriched droplets-liposomes as with other raft-associated proteins [43]. These findings suggest that flotillins depend on a wide range of cell types for subcellular localization which is highly dynamic and active during the endocytosis process. In our study, SpFLT-1 was not only localized in the plasma membrane but likely expressed in the intracellular vesicles of the hemocytes, as evidenced from the observation using OptiPrep fractionation assays in which the SpFLT-1 protein was found distributed at the middle layers 5, 6 and 7 (Fig 4C). The reversible palmitoylation process of flotillin-1 means that it possesses more diverse positions in which it could freely shuttle between the plasma membrane and various intracellular organelles [44].

Some studies show that flotillin-1 may play a key role in the early developmental stage of zebrafish and the fruit fly. Knockdown of flotillins from developing zebrafish *in vivo* results in the occurrence of very serious morphological defects in early gastrulation, indicating the basic and ubiquitous functions of flotillins involved in neural development [45]. Flotillins also show high expression level in the nervous system in early development stages of *D*. *melanogaster* [46]. Similarly, SpFLT-1 was highly expressed in the early embryonic stages in our study, suggesting that this gene may exert its function from the early development of *S*. *paramamosain*. Since SpFLT-1 was also highly expressed in both maternal ovaries and the zygote stage of the embryo, we wondered whether the SpFLT-1 gene might involve maternal transfer. This phenomenon is observed in mammals, fishes and also in crustaceans [47–49].

The cell membrane is the first line of defense to prevent extracellular material especially pathogenic microorganisms (such as bacteria, viruses, etc.) freely entering the cell and guarantees a relatively stable intracellular environment. Conversely, various pathogens have developed corresponding escape mechanisms against the host’s immune system. They can take advantage of different membrane structures or some receptors in the host cell membranes to enter cells and cause infection. There are three main pathways which are now confirmed for pathogenic internalization occurring at the plasma membrane of mammalian cells: phagocytosis, clathrin-mediated endocytosis and non-clathrin-mediated uptake occurring at lipid rafts [50, 51].

The endocytic machinery plays a crucial role in the process of pathogen internalization. Endocytosis of small size pathogens is always mediated by specific single endocytosis pathways. For instance, bacterial toxins, such as anthrax originally from *Bacillus anthraci* [52] and tetanus from *Clostridium tetani* [53], can easily enter into clathrin-coated vesicles; while tetanus [54] enters cells through the lipid raft caveolae. A bacterium, even though it is larger than a virus particle, in addition to phagocytosis, can also recruit clathrin, caveolin and other cell-surface receptor to achieve the purpose of internalization. *Listeria monocytogenes* colocalizes with factor receptor Met and HGF, ubiquitin ligase Cbl, clathrin and dynamin during entry into mammalian cells [24]. It is confirmed that type 1 fimbriated *Escherichia coli* uroepithelial invasion occurs through lipid rafts, which provides valuable reference for disease therapy [23]. Our present results indicated that the silencing of SpFLT-1 inhibited the endocytosis rate of *V*. *alginolyticus* in hemocytes of *S*. *paramamosain* while its overexpression could facilitate *V*. *alginolyticus* entry into the EPC cells. Correspondingly, *Vibrio* protein was in the same fraction layer as SpFLT-1 protein at 3 hpi in hemocytes and it showed high expression signals when SpFLT-1 was dramatically up-regulated in gills at 3 hpi, which indicated that they may interact with each other or exhibit a close cross link. In view of our results, it would be presumed that SpFLT-1 had a potential role in the process of bacterial infection. The same phenomenon is reported in an earlier study that flotillin-1 colocalizes with the inclusion membrane protein A in the *C*. *pneumoniae* inclusion membranes in the human line and A549 epithelial cell lines [19].

Flotillins mediating endocytosis are clathrin-independent and the associated membrane curvature is different from the caveolae [28]. The endocytic process either depends on dynamin [55] or non-dynamin absorption [56]. Recent studies show that the membrane protein NPC1L1, APP and dopamine transporter enter the cells through clathrin vesicles, and that flotillins are required [57]. This indicates that flotillin microdomains could recruit some specific molecules to enhance clathrin-mediated endocytosis efficiency. However, there is no direct evidence to confirm the interaction between flotillins and clathrin. In *S*. *paramamosain*, whether the entry of *V*. *alginolyticus* into the hemocytes and epithelium cells of gills is mediated by clathrin or flotillin microdomains or depends on multiple endocytic proteins as discussed above remains to be further investigated.

It is worthwhile mentioning that besides endocytosis, flotillins may also have an activity in interaction with intracellular pathogens. Thus, flotillins are required for cholera toxin trafficking but are not involved in the endocytosis of the toxin which may be mediated by other endocytic pathways [13]. Other studies also show that the intracellular transport of Shiga and ricin toxin also relies on flotillins and knockdown of flotillins does not affect the absorption of these two toxins but strengthens their toxicity [14]. However, whether SpFLT-1 would be responsible for the intracellular transport of bacterial toxin remains unknown in the present study and further investigation should be performed to address this.

The phagocytic process can protect cells from infection and plays a crucial role in the innate immune system. However, pathogens may utilize the phagocytosis mechanisms to facilitate their internalization process [58]. They can prevent the phagosome maturation and movement and weaken its bactericidal activity. Besides, some bacteria may escape from the vesicle and replicate in the cytoplasm. An earlier study also notes that flotillin-1 is enriched in the mature phagosome [20], which is gradually raised in the process of phagosome maturation other than from the plasma membrane. In our study, SpFLT-1 was distributed in the middle layers of the hemocytes, which implied that it might be located in intracellular vesicles, such as the phagosome. Further study in A549 epithelial cell lines shows that flotillin-1 protein associated with the inclusion membrane protein A in *C*. *pneumoniae* contributes to intracellular bacteria growth [19]. Therefore, whether the crab SpFLT-1 could play a specific function in the process of the formation of phagolysosomes or prevent or contribute to the growth of intracellular pathogens remains inconclusive and needs further study.

In conclusion, the present study presented a new flotillin-1 homologous gene SpFLT-1 identified in *S*. *paramamosain*, and its cDNA sequence and DNA organization were determined. As with the other reported flotillin-1, SpFLT-1 expression in tissues and its localization in cells showed its potential role related to endocytosis of the tested bacterium *V*. *alginolyticus*, suggesting that SpFLT-1 may act as a key protein during the process of bacterial infection. This is the first report of a new SpFLT-1 which may be associated with endocytosis in marine invertebrates including crabs, but the clear molecular function of SpFLT-1 in *V*. *alginolyticus* internalization remains to be further elucidated in future studies.

## Material and Methods

### Animals, bacterial challenge, sample collection and mRNA transcript analysis

Live healthy *S*. *paramamosain* (300 ± 50 g in weight) purchased from a local commercial crab farm were acclimated at 25 ± 2°C for one week before the experiments were carried out.

Crabs were randomly divided into two groups. The experimental group including 30 crabs were challenged with injection of 100 μL live pathogenic bacteria (*V*. *alginolyticus*, CGMCC 1.1833) (3.6×10^7^ CFU/mL) in the modified crab saline solution (NaCl, 496 mM; KCl, 9.52 mM; MgSO_4_, 12.8 mM; CaCl_2_, 16.2 mM; MgCl_2_, 0.84 mM; NaHCO_3_, 5.95 mM; HEPES, 20 mM; pH 7.4) into the base of the right fourth leg. The other 30 individuals were injected with 100 μL sterile saline solution as the control group. Sampling was performed at different time intervals (3, 6, 12, 24, 48 and 96 h) (n = 5) after bacterial challenge. Hemocytes were isolated from the haemolymph as described previously [7]. Samples from the hemocytes and the gills were separately collected from each individual animal and were frozen immediately in liquid nitrogen, and stored at– 80°C for SpFLT-1 mRNA and protein expression pattern analysis.

For the SpFLT-1 tissue distribution study, the hemocytes, ovaries, midgut gland, thoracic ganglion mass, gills, heart, spermathecae, muscle, stomach, eyestalk, brain, reproductive tract, hepatopancreas and subcuticular epithelia were sampled (n = 5), frozen individually immediately in liquid nitrogen, and stored at– 80°C for later use. Hemocytes and gills in this group were chosen for immunofluorescence assay. Maternal ovaries, zygote, early to late-staged embryos and the zoea 1 stage larva of *S*. *paramamosain* were sampled as in our previous study [5].

Total RNA of the different tissues of individuals were extracted using Trizol Reagent (Life Technologies) following the manufacturer's instructions and the cDNA template was synthesized using a PrimeScript RT reagent kit (Perfect Real Time) (Takara). qPCR quantification and data analysis were performed as described previously [59]. The gene-specific primers are shown in S1 Table. The β-actin gene was employed as the internal standard in the SpFLT-1 gene tissue distribution study and the GAPDH gene for the bacterial challenge experiment.

### Determination of SpFLT-1 cDNA and genomic DNA sequence

RACE 5′ and 3′ were carried out to obtain the full-length cDNA of SpFLT-1. Specific primers were designed based on the obtained partial cDNA sequence from an SSH cDNA library as shown in S1 Table. The PCR amplification was performed as described previously [7].

Genomic DNA was extracted from the muscle of *S*. *paramamosain* using a Universal Genomic DNA Extraction Kit (Takara) following the manufacturer′s instructions. Based on the full-length cDNA sequence of SpFLT-1 obtained, six pairs of primers (listed in S1 Table) were designed. The amplification conditions were 5 min at 94°C; 35 cycles of 10 s at 98°C, 8 min at 68°C, followed by an extension at 72°C for 10 min.

Purification, ligation, transformation, identification, and sequencing of the amplification products were performed and the sequences obtained were analyzed as described previously [7]. Multiple sequence alignment was carried out with Clustal X 1.83 software. The neighbor-joining method was used to reconstruct a phylogenetic tree with 1000 bootstrap replicates using MAGA v4.0 software. The isoelectric point of the polypeptide was predicted using DNAsist 2.0 software.

### Antibody preparation

The whole coding sequence of SpFLT-1 was inserted into the expression vector pET-28(a+) (Novagen) through the restriction enzyme recognition sites of *Nco* I and *Xho* I. The inserted sequence was amplified using specific primers orFLT1-F/orFLT1-R and primers pFLT1-F/pFLT1-R (S1 Table). The protocol of construction and expression of recombinant SpFLT-1 was as previously described [60]. The recombinant SpFLT-1 was purified in gel slices and it was further confirmed using matrix assisted laser desorption ionization-time of flight mass spectrometry also as described previously [61].

To obtain a polyclonal antibody against SpFLT-1, the purified recombinant protein was used as an antigen to immunize BALB/C mice for more than one month until the level of antibody in their sera reached the titer required for experimentation. The immunization serum was purified using Nab Spin Kit, 1 mL for Antibody Purification (Thermo Scientific) following the manufacturer′s instructions. The titer, purity and specificity of the purified anti-SpFLT-1 antibody were analyzed using an ultraviolet spectrophotometer, ELISA, SDS-PAGE and immune-blotting.

The primary antibodies used in the immune-blotting experiments were as follows: mouse anti-β-actin antibody (ZSGB-BIO); mouse anti-GAPDH antibody (ZSGB-BIO); rabbit anti-HA antibody (ZSGB-BIO); and rabbit anti-*Vibrio* species (Gaithersburg, MD). Goat anti-mouse IgG horseradish peroxidase (HRP)-conjugated (ZSGB-BIO) and goat anti-rabbit IgG HRP-conjugated (ZSGB-BIO) antibody were used to recognize the primary antibodies. The fluorescence-conjugated secondary antibodies were goat anti-mouse IgG (Alexa Fuluor 647) (Abcam) and goat anti-rabbit IgG FITC conjugated (Multiscience).

### Triton X-100 extraction, OptiPrep flotation and fractionation

To extract DRMs from the infected crabs, the hemocytes collected and the gills ground to powder were kept on ice and lysed with 1 mL of ice-cold 1% Triton X-100 in base buffer (20 mM Tris-HCl, 250 mM sucrose, 1 mM CaCl_2_, 1 mM MgCl_2_, pH7.8) containing protease inhibitor cocktail (Roche) for 30 min [62]. The cells were then homogenized by passing 50 times through a 1mL syringe. The lysates were centrifuged at 1,000 g for 10 min, and the resulting supernatant was collected and transferred to a separate tube. The protein concentrations were determined using a Pierce BCA Protein Assay Kit (Thermo Scientific) with bovine serum albumin as the standard. Equal protein amounts were subjected to an OptiPrep gradient. Preparation of the OptiPrep (Sigma) density gradient layers and isolation of DRMs were conducted following the manufacturer′s instructions using a Caveolae/Raft Isolation Kit (Sigma). After centrifugation in an S52ST rotor (Hitachi CS150GXL) at 200,000 g at 4°C for 4 h, nine fractions were collected from the top of the gradient.

### Protein extraction and immune-blotting

Various tissues collected from normal crabs and the gills collected from the bacterially challenged crabs were ground to powder and then lysed with RIPA lysis buffer (Beyotime Biotechnology) and protease inhibitor cocktail (Roche) for 30 min. The protein concentrations of the lysates were detected as described above. Proteins from equal volumes of OptiPrep gradient fractions were precipitated using trichloroacetic acid. The concentrated supernatants and lysates from the tissues were re-suspended in SDS sample buffer. For immune-blotting analysis, protein samples in the SDS sample buffer were boiled for 10 min and separated using SDS-PAGE. The blotted proteins were blocked using 5% nonfat milk, probed with primary antibodies, followed by HRP-conjugated secondary antibodies. The *Vibrio* protein was detected using dot-blotting aliquots from each fraction on PVDF membrane (BIO-RAD). Signals were detected using an enhanced chemiluminescence Western blotting kit (Millipore Corporation).

### RNA interference assays

The DNA fragments (~ 500 bp in length) containing coding sequences for the proteins to be ′knocked down′ were amplified using PCR. Each primer used in the PCR contained a 5′-T7 RNA polymerase binding site (5′-TAATACGACTC ACTATAGGG-3′) followed by sequences specific for the targeted genes (S1 Table). The control templates were produced using PCR with primers specific to one part of the green fluorescent protein gene from the pEGFP-1 vector (Clontech). The PCR products were purified and used as templates using a Megascript T7 Transcription Kit (Ambion) to produce double-stranded RNA (dsRNA) following the manufacturer’s protocol. The dsRNAs were purified using the lithium chloride precipitation method.

Hemocytes from the *S*. *paramamosain* (2×10^6^ cells/mL) were plated in 96-well plates in Leibovitz L-15 medium with 5% fetal bovine serum (FBS, Gibco) 1d prior to transfection at 90% confluence at 20°C [63]. Transfections were carried out with Lipofectamine 2000 (Life Technologies) to a final concentration of 10 μg/mL dsRNA following the manufacturer’s recommendations. The cells were incubated for 48 h and, then, the RNA and protein samples were collected for SpFLT-1 expression analysis using semi-quantitative PCR and immune-blotting, and the cells in which SpFLT-1 protein showed effective interference were subjected to endocytosis assays.

### Expression of SpFLT-1 in EPC cells

pCMV-HA-FLT1 was generated by subcloning the PCR-amplified SpFLT-1 coding region sequence into a pCMV-HA vector (Clontech). The EPC cells were kindly provided by Professor Xinhua Chen (Third Institute of Oceanography, State Oceanic Administration, China). The cells were seeded in Leibovitz L-15 medium (Hyclone) containing 10% FBS, penicillin (100 μg/mL) (Gibco), and streptomycin (100 μg/mL) (Gibco) in a 48-well plate at 25°C. After being cultured for 24 h, transfection of the plasmid was performed using Lipofectamine 2000 reagent. Stable cell lines expressing SpFLT-1 were selected for endocytosis and immunofluorescence assay.

### Endocytosis assay

Fluorescein isothiocyanate (FITC)-conjugated heat-killed *V*. *alginolyticus* was prepared using a modified method described previously [64]. Briefly, the bacteria were heated for 60 min at 70°C in a water bath and then washed three times by centrifuging for 20 min at 900 g and 4°C. The bacteria were then incubated at a concentration of 10^9^ cells/mL in 0.1 M Na_2_CO_3_ (pH 9.5), containing 0.1 mg/mL FITC (Sigma) for 30 min at 37°C. After that, the bacteria were washed five times in PBS at 12,000 g for 3 min, resuspended at a concentration of 10^8^ cells/mL in PBS, and stored at– 20°C until use.

The dsRNA and pCMV-HA-FLT1 transfection assays were performed as described above, and about 2 × 10^7^ cells/mL FITC-conjugated heat-killed *V*. *alginolyticus* was added to the cell medium. Meanwhile, hemocytes and EPC cell incubated in 4°C were used as control. After incubation for 2 h, the cells were fixed with 4% paraformaldehyde in PBS at pH 7.4 and were then harvested from the plates to a new tube and centrifuged for 8 min at 250 g. They were washed more than four times to eliminate most residual extracellular bacteria and then resuspended at 10^6^ cells/mL, attached onto glass slides and stained with DAPI (0.5 μg/mL). The fluorescence of the adhering FITC-conjugated bacteria was quenched by adding a few drops of 0.4% trypan blue (Sigma) for several minutes and then replaced with 0.15 M NaCl. The ingested bacteria were easily detected under confocal microscopy (LSM780, Zeiss). The endocytosis rates of the cells was determined by counting at least a million cells on each slide in three parallel samples using an iCys quantitative image cytometer (Beckman) and dividing the number of cells with ingested fluorescent bacteria by the total number of counted cells. Data from three independent experiments were analyzed.

### Immunofluorescence assay

Gills from the normal group of crabs were chosen to measure the tissue distribution of SpFLT-1 protein using immunofluorescence assay. The gills were fixed with 4% paraformaldehyde after sampling. Six micrometer thick paraffin wax embedded gill sections were deparaffinized and rehydrated using routine protocols and heated with citrate buffer (0.01 M, pH 6.0) in a microwave oven for 3 min. They were then incubated in 3% hydrogen peroxide in methyl alcohol for 20 min to block the endogenous peroxidase activity. The sections were then incubated with the blocking buffer (1% BSA/0.3 M glycine/10% normal goat serum (Solarbio) in 0.1% PBS-Tween) for 1 h to block non-specific protein-protein interactions, which was followed by incubation with mouse polyclonal anti-SpFLT-1 antibody (1:500 dilutions) and pre-immune serum as control for 1 h. The sections were then incubated with goat anti-mouse IgG (Alexa Fuluor 647) (1:1000 dilutions) for 45 min. DAPI was used to stain the cell nuclei. After the hemocytes from normal group crabs were fixed with 4% paraformaldehyde, they were attached onto glass slides. The steps were conducted as described above.

EPC cells expressing SpFLT-1 were also fixed with 4% paraformaldehyde and then incubated with the blocking buffer for 1 h. The cells were then incubated with rabbit anti-HA antibody overnight at 4°C and the secondary antibody was goat anti-rabbit IgG FITC conjugated.

All the samples were examined using confocal microscopy.

### Statistical analysis

Analysis of variance comparison tests were used for statistical analysis using SPSS software (version 11.5). Data are shown as mean ± S.E. Differences were considered to be significant when *p* < 0.05 (*), or *p* < 0.01 (**).

## Supporting Information

S1 Fig Prokaryotic expression and purification of SpFLT-1 and its antibody preparation. (A) SDS—PAGE analysis of pET-28a/SpFLT-1 expression. (B) Dissolubility analysis of recombinant SpFLT-1. (C) Purification of recombinant SpFLT-1. (D) Purity analysis of prepared SpFLT-1 antibody. (E) Titer analysis of prepared SpFLT-1 antibody. (F) Detection specificity of prepared SpFLT-1 antibody. Lane 1: pET-28a(+) vector only; lane 2: recombinant SpFLT-1; lane 3: sonication supernatant; lane 4: sonication precipitation; lane 5: purified recombinant SpFLT-1; lane 6: prepared SpFLT-1 antibody; and lane 7: SpFLT-1 protein detected in hemocytes of *S*. *paramamosain* by western blot.
(TIF)

S1 Table Sequences of primers used in this study. (DOCX)

S2 Table Homology analysis among SpFLT-1 and the flotillin-1 of other species. (DOCX)
