# Supplementary figures and images for: A New Membrane Lipid Raft Gene SpFLT-1 Facilitating the Endocytosis of Vibrio alginolyticus in the Crab Scylla paramamosain
Source: PLoS One. 2015 Jul 17;10(7):e0133443. doi: 10.1371/journal.pone.0133443 (PMC4506021; doi:10.1371/journal.pone.0133443)

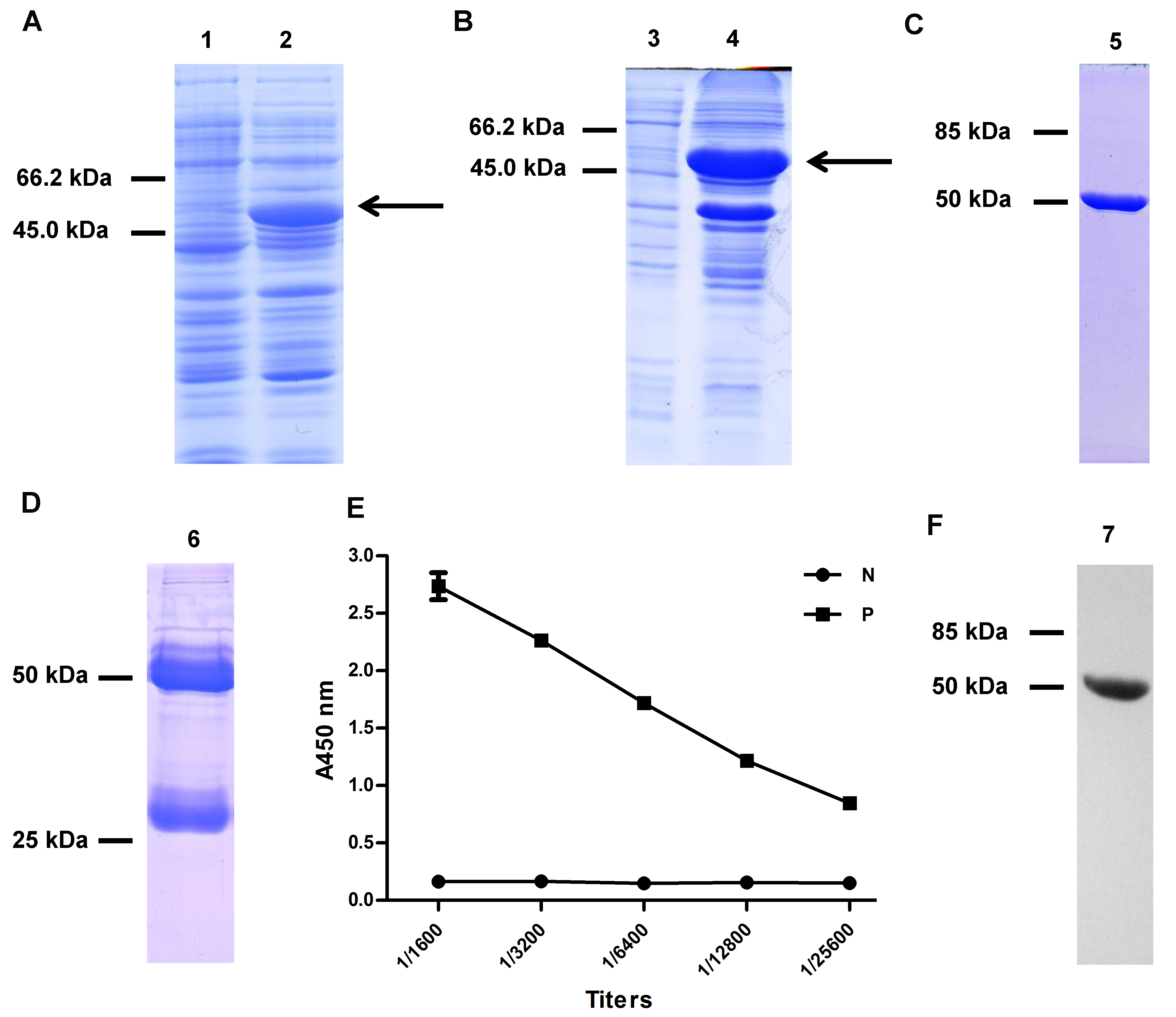

Supplement: S1 Fig — (A) SDS—PAGE analysis of pET-28a/SpFLT-1 expression. (B) Dissolubility analysis of recombinant SpFLT-1. (C) Purification of recombinant SpFLT-1. (D) Purity analysis of prepared SpFLT-1 antibody. (E) Titer analysis of prepared SpFLT-1 antibody. (F) Detection specificity of prepared SpFLT-1 antibody. Lane 1: pET-28a(+) vector only; lane 2: recombinant SpFLT-1; lane 3: sonication supernatant; lane 4: sonication precipitation; lane 5: purified recombinant SpFLT-1; lane 6: prepared SpFLT-1 antibody; and lane 7: SpFLT-1 protein detected in hemocytes of S. paramamosain by western blot. (TIF) [file pone.0133443.s001.tif]
