# Supplementary material for: A New Membrane Lipid Raft Gene SpFLT-1 Facilitating the Endocytosis of Vibrio alginolyticus in the Crab Scylla paramamosain
Source: PLoS One. 2015 Jul 17;10(7):e0133443. doi: 10.1371/journal.pone.0133443 (PMC4506021; doi:10.1371/journal.pone.0133443)
Supplement: S1 Table — (DOCX) [file pone.0133443.s002.docx]

| Primers | | Sequences(5'-3') | |
| --- | --- | --- | --- |
| **5′ RACE** | |  | |
| 5FLT-R | | TTTCTCGGAGTCAGCAGGTTGTTTA | |
| **3′ RACE** | |  | |
| 3FLT-F | | GGCAAACAAGGACTCCCAAATCAAG | |
| **Genomic DNA** | | | |
| FLTi1-F | | GGACTTGTCACCTGTGGCCCAAATGAG | |
| FLTi1-R | | ACCTCGACGGACATTGACCCCATGAT | |
| FLTi2-F | | TGCGAGCAGTTTTTGGGCAAGACA | |
| FLTi2-R | | CTTCTTGCGGTCTTTGTAGATTTCCT | |
| FLTi3-F | | ATGTCCGTCGAGGTGAGCATGAGGAC | |
| FLT3-R | | GAAGCCGCACATACCTTAGCCTGCAC | |
| FLTi4-F | | GAGGAGAGGATGGCTGCTAAATATG | |
| FLTi4-R | | CTCCTCTGAATCTCTTGCTCTTGAAT | |
| FLTi5-F | | AAACAACCTGCTGACTCCGAGAAAT | |
| FLTi5-R | | GACCTTCGGCAGGGTTGACAG | |
| FLTi6-F | | AGAAGCTCGCAGAGGCAGCCCGGAAC | |
| FLTi6-R | | CATGACCTCCCCAGTCAGCTTCGAT | |
| **qPCR** | |  | |
| rt-FLT1-F | | CGTGTTTGAAGTGGCGTCCTCCGAT | |
| rt-FLT1-R | | TGTTTGCCTCAGCCTCGCCAATCCT | |
| rt-actin-F | | GCCCTTCCTCACGCTATCCT | |
| rt-actin-R | | GCGGCAGTGGTCATCTCCT | |
| rt-GAPDH-F | | CTCCACTGGTGCCGCTAAGGCTGTA | |
| rt-GAPDH-R | | CAAGTCAGGTCAACCACGGACACAT | |
| **Recombination expression** | | | |
| orFLT1-F | | ATGGTGTGGGGACTTGTCACCTGTG | |
| orFLT1-R | | AACGGCACGGATTGTCTTGGACACG | |
| pFLT1-F | | TTCCATGGTGTGGGGACTTGTCACC | |
| pFLT1-R | | AGACTCGAGAACGGCACGGATTGTCTT | |
| **dsRNA synthesis** | | | |
| T7-#1 FLT1-F | | TAATACGACTCACTATAGGGGAGGCTGAGGCAAACAAAGACTCCC | |
| T7-#1 FLT1-R | | TAATACGACTCACTATAGGGCCTGCAGAGCGGCCGCCTTCTG | |
| T7-#2 FLT1-F | | TAATACGACTCACTATAGGGTAAGGTGGTGGAACGCGCCCAGGAA | |
| T7-#2 FLT1-R | | TAATACGACTCACTATAGGGACGGCACGGATTGTCTTGGACACGT | |
| T7-GFP-F | | TAATACGACTCACTATAGGGATGGTGAGCAAGGGCGAGGAGC | |
| T7-GFP-R | TAATACGACTCACTATAGGGTCTTGAAGTTCACCTTGATGCC | |  |
| **RNAi efficiency detection** | | |  |
| ef-FLT1-F | TAAGGTGGTGGAACGCGCCCAGGAA | |  |
| ef-FLT1-R | GTCCATTTCTTCTCAATCTTCCTCT | |  |
| **SpFLT-1 overexpression** | | |  |
| pCMV-FLT1-F | CGGAATTCCAGTGTGGGGACTTGTCACCTGTG | |  |
| pCMV-FLT1-R | CCGCTCGAGCTAGAGATTAACGGCACGGATTG | |  |
