# Supplementary material for: A New Membrane Lipid Raft Gene SpFLT-1 Facilitating the Endocytosis of Vibrio alginolyticus in the Crab Scylla paramamosain
Source: PLoS One. 2015 Jul 17;10(7):e0133443. doi: 10.1371/journal.pone.0133443 (PMC4506021; doi:10.1371/journal.pone.0133443)
Supplement: S2 Table — (DOCX) [file pone.0133443.s003.docx]

| Species | GenBank accession number | Identity with SpFLT-1 (%) |
| --- | --- | --- |
| *Drosophila melanogaster* (fruit fly) | NP_477358 | 74 |
| *Apis mellifera* (honey bee) | XP_623738 | 74 |
| *Aedes aegypti* (yellow fever mosquito) | XP_001651132 | 75 |
| *Bombus impatiens* (common eastern bumble bee) | XP_003484724 | 74 |
| *Danaus plexippus* (monarch butterfly) | EHJ74021 | 75 |
| *Acromyrmex echinatior* (Panamanian leafcutter ant) | EGI57731 | 74 |
| *Metaseiulus occidentalis* (western predatory mite) | XP_003747935 | 69 |
| *Ciona intestinalis* (vase tunicate) | XP_002123705 | 66 |
| *Oryzias dancena* (brackish medaka) | ACN49164 | 63 |
| *Danio rerio* (zebrafish) | NP_570988 | 63 |
| *Xenopus laevis* (African clawed frog) | NP_001082374 | 65 |
| *Takifugu rubripes* (Fugu rubripes) | NP_001092129 | 64 |
| *Salmo salar* (Atlantic salmon) | ACN10783 | 62 |
| *Homo sapiens* (human) | AAD40192 | 62 |
| *Mus musculus* (house mouse) | NP_032053 | 62 |
| *Bos taurus* (cattle) | AAI04517 | 61 |
